# The sialyl-glycolipid SSEA4 marks a subpopulation of chemotherapy resistant breast cancer cells with mesenchymal features

**DOI:** 10.1186/2051-1426-3-S1-O1

**Published:** 2015-08-14

**Authors:** Andrea Aloia, Evgeniya Petrova, David Agorku, Annalisa Terranegra, Alessandra Mingione, Jean-Gabriel Judde, Andreas Bosio, Stefano Cairo, Olaf Hardt

**Affiliations:** 1 Institute for Molecular Health Sciences, ETH Zurich, Switzerland; 2 Miltenyi Biotec GmbH, Bergisch Gladbach, Germany; 3 R&D, Xentech SAS, Institute Pasteur, Evry, France; 4 R&D, Miltenyi Biotec GmbH, Bergisch Gladbach, Germany; 5 Investigator Nutrigenetics, Sidra Medical and Research Center, Doha, Qatar; 6 Department of Health Sciences, University of Milan, Milan, Italy; 7 XenTech SAS, Evry, France

## 

Triple negative breast cancer (TNBC) is an aggressive breast cancer subtype associated with high risk of early relapse and metastasis [1]. At the moment chemotherapy remains the main option for systemic therapy of TNBC patients but complete remission occurs only in 20% of the patients [2]. In order to identify biomarker for chemotherapy-resistant TNBC cells, we performed a cell surface marker screen in 4 TNBC patient-derived xenograft (PDX) models that respond well to adriamycin/cyclophosphamide-based (A/C) chemotherapy but fail to reach complete pathological response. We used multi-parameter flow cytometry to screen the expression of 45 cell surface markers during the course of chemotherapy.

We identified the sialyl-glycolipid SSEA4 as a marker of chemotherapy-resistant cancer cells in all four models. In addition, 3 out of 4 TNBC PDXs showed higher percentage of SSEA4-positive cells compared to all A/C-sensitive TNBC PDXs analysed. Gene expression comparison between SSEA4-positive and SSEA4-negative tumor cells from 3 TNBC PDXs highlighted an overexpression of mesenchymal-associated genes and a deregulation of drug resistance pathway-associated genes and miRNAs in SSEA4+ breast cancer cells. In addition, high expression of ST3 beta-galactoside alpha-2,3-sialyltransferase 2 (ST3Gal2), the enzyme catalyzing the last step of SSEA4 synthesis, was found associated with poor outcome in ER-, PR- breast cancer patients treated with chemotherapy (p < 0.01, HR 3.08).

Thus, we propose SSEA4 as a novel marker of mesenchymal and chemoresistant breast cancer cells, and ST3GAL2 expression as a predictive marker for chemoresistance associated with poor outcome in breast cancer patients.
